# A modelling paradigm for RNA virus assembly

**DOI:** 10.1016/j.coviro.2018.07.003

**Published:** 2018-08

**Authors:** Reidun Twarock, Richard J Bingham, Eric C Dykeman, Peter G Stockley

**Affiliations:** 1 York Centre for Cross-disciplinary Systems Analysis, University of York, York YO10 5GE, UK; 2 Department of Mathematics, University of York, York YO10 5DD, UK; 3 Department of Biology, University of York, York YO10 5NG, UK; 4 Astbury Centre for Structural Molecular Biology, University of Leeds, Leeds LS2 9JT UK

## Abstract

Virus assembly, a key stage in any viral life cycle, had long been considered to be primarily driven by protein–protein interactions and nonspecific interactions between genomic RNA and capsid protein. We review here a modelling paradigm for RNA virus assembly that illustrates the crucial roles of multiple dispersed, specific interactions between viral genomes and coat proteins in capsid assembly. The model reveals how multiple sequence-structure motifs in the genomic RNA, termed packaging signals, with a shared coat protein recognition motif enable viruses to overcome a viral assembly-equivalent of Levinthal’s Paradox in protein folding. The fitness advantages conferred by this mechanism suggest that it should be widespread in viruses, opening up new perspectives on viral evolution and anti-viral therapy.

## Highlights

• Multiple, common dispersed packaging signals (PSs) promote RNA virus assembly.
• PS-mediated assembly solves a viral equivalent of Levinthal’s Paradox.
• RNA PSs occur in many viral families, including Picornaviruses and Hepatitis B virus.
• PSs encode an assembly manual that can be repurposed for VLP production.

**Current Opinion in Virology** 2018, **31**:74–81

This review comes from a themed issue on **Virus structure and expression**

Edited by **Kay Grünewald** and **Thomas Krey**

For a complete overview see the Issue and the Editorial

Available online 2nd August 2018

**https://doi.org/10.1016/j.coviro.2018.07.003**

1879-6257/© 2018 The Authors. Published by Elsevier B.V. This is an open access article under the CC BY license (http://creativecommons.org/licenses/by/4.0/).

## Introduction

The formation of a viral protein container encapsulating a virus’ genomic cargo is a prerequisite for the successful propagation of a viral infection. A better understanding of this process can therefore be exploited for therapy, either via the development of antiviral strategies inhibiting assembly, or the repurposing of the self-assembly process for the design of gene vectors and vaccines.

The initial focus in the study of virion assembly was directed towards *in vitro* studies of capsid self-assembly in the absence of other viral components. Models developed in tandem with such experiments provided an understanding of the kinetics [1, 2, 3] and thermodynamics [4,5] of spontaneous capsid self-assembly, and of the roles of protein–protein interactions in defining quasiequivalent capsid geometries [6,7]. They also elucidated the local rules underpinning coat protein (CP) self-association during capsid formation [8,9]. Many viruses, especially double-stranded DNA viruses, assemble their capsids prior to genome packaging via an ATP driven packaging motor. The protein-centric models, with the addition of scaffolding proteins in the case of larger capsid shells, are therefore an adequate context to study capsid assembly in these cases. By contrast, single-stranded RNA viruses, the largest family of viruses and containing many important human pathogens, package their genomes during capsid assembly, exhibiting a co-assembly process. For these viruses, capsid assembly has to be modelled in tandem with genome packaging. An important aspect of virus assembly in the presence of genomic RNA is the need for genome compaction [10], and several groups have made important contributions to the modelling of this aspect of virus assembly [11^•^,^12^,13^•^,14^•^]. The impact of non-specific electrostatic interactions between genomic RNAs and CP [15, 16, 17, 18,19^••^] and of the stiffness of the RNA molecule on the assembly process [20^•^] have been analysed. It has also been shown that the secondary structure of the RNA molecules play an essential role in determining capsid morphology in the self-assembly of Cowpea Chlorotic Mottle Virus (CCMV)-like particles [21]. The roles of genomic RNA have been studied in the assembly of helical viruses [22^••^]. Moreover, molecular dynamics simulations of capsid assembly, both in the absence and presence of different types of cargoes, have made important contributions to our understanding of virus assembly [23^••^,^24^]. Indeed, viral capsids can be assembled *in vitro* around different types of cargoes, including anions [25, 26, 27]. The models presented here go one step further. Instead of viewing viral genomes as passive passengers with at most non-specific electrostatic contributions to the assembly process, they demonstrate the consequences of the cooperative action of multiple, sequence-specific contacts between genomic RNA and CP.

## Genomic RNA is not a passive passenger

**Figure 1:**
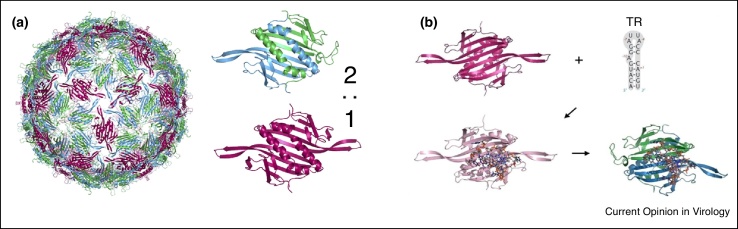
Genomic RNA defines capsomer conformation in MS2. **(a)** The MS2 capsid (based on pdb-id 1ZDH) is formed from asymmetric (blue/green) and symmetric (pink) forms of the coat protein dimer in a 2:1 ratio. **(b)** The stem–loop TR triggers a conformational change from the symmetric to the asymmetric form of the coat protein dimer. The characteristic packaging signal recognition motif is given by (x)xYA in the apical loop of the stem–loop, and the A in the 5′ bulge. Other stem–loops in the viral genome sharing aspects of this motif can also function as packaging signals [29,32].

Even in the absence of the genomic RNA, the CP of most single-stranded RNA viruses can self-assemble *in vitro*, but the process is typically much faster and more efficient in the presence of genomic RNA. This is the case, for example, for the assembly of the MS2 capsid (Figure 1) in the presence of multiple copies of the translational repressor (TR) [28], a stem–loop in the genomic RNA known to function also as a packaging signal. This observation suggests that the contributions from genomic RNA to the assembly process are significant and therefore cannot be neglected in the assembly models.

There is only one copy of TR in the MS2 genome. Binding of TR to the CP dimer triggers a conformational switch from the symmetric dimer, the dominant form in solution, to its asymmetric conformation [29] that is needed in a 2:1 ratio for the construction of the capsid (Figure 1a). Normal mode analysis has revealed the structural features of TR that are required for this allosteric effect [30,31], demonstrating that many other, multiple dispersed, stem–loops in the MS2 genome could trigger the same effect [32]. This has resulted in the packaging signal (PS) hypothesis: Multiple dispersed secondary structure elements in the genomic RNA, with CP recognition features akin to those of the known high affinity PS, also trigger conformational changes of the CP dimer to its asymmetric conformation. These multiple dispersed sites have been called PSs, in analogy to the high affinity PS with which they share their characteristic feature for CP recognition. In the case of MS2, assembly mediated by these multiple dispersed PSs is also known as the dimer-switching model [33]. In other viruses, PSs can play a number of different roles in promoting capsid formation [35^••^,36^•^,45]. However, these different scenarios all share the same basic mechanism of PS-mediated assembly, in which multiple dispersed sites in the (pre)genomic viral RNA with affinity for CP promote efficient formation of a viral capsid with the correct geometry.

## A mathematical model of PS-mediated assembly

**Figure 2:**
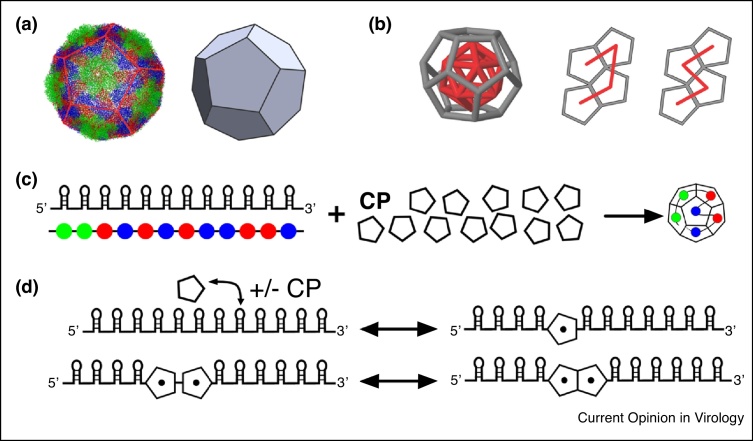
A modelling paradigm for packaging signal-mediated assembly. **(a)** A dodecahedral model system is used as a coarse-grained representation of capsid geometry. **(b)** The order in which the protein building blocks of the capsid (pentamers) are recruited is indicated by a connected line (path) that connects midpoints of adjacent pentamers. A connected subset of such a path is shown superimposed on capsid assembly intermediates formed from four pentamers; the two examples represent different assembly scenarios. **(c)** The assembly of the dodecahedral model system from 12 pentamers is modelled in the presence of RNAs, that are represented by 12 beads, each of which represents a PS. Beads are colour-coded according to their affinities for CP, as green (strong), blue (intermediate) and red (weak). **(d)** The system assembles based on a set of local rules that are formulated as assembly reactions, describing RNA:CP and CP:CP interactions.

In order to investigate how such multiple dispersed PS sites mediate capsid assembly, we developed a mathematical model that captures their collective impact on virus assembly efficiency (Figure 2) [37,38^••^]. From a geometric point of view, the simplest model of an icosahedral capsid is a dodecahedral shell formed from 12 pentagonal capsid building blocks (pentamers). This is representative of small plant viruses (*T* = 1 geometries in the Caspar–Klug classification [39]), or the structures of Picornaviruses (*(Pseudo)T* = 3 structures in which pentamers are formed by five protomers, each consisting of different polypeptides corresponding to the structural protein (VP) units). The model captures the assembly of 12 pentamers into a dodecahedral shell according to a set of simple local rules (Figure 2b): pentamers associate with, and disassociate from, PSs on the genomic RNA with rates depending on CP:PS affinity. As the precise nucleotide sequences of the PSs vary around their shared recognition motif, their affinities for CP can be distinct. In our model, they fall into three categories, weak (from 0 to −4 kcal/M), intermediate (from −4 kcal/M to −8 kcal/M), and strong (from −8 kcal/M to −12 kcal/M), reflecting affinities seen in MS2 [40,41]. If two pentamers are bound to adjacent PSs, they form (or subsequently break) CP—CP interactions with rates determined by the free energy of the CP:CP bonds, chosen to be −2.5 kcal/M following estimates in Ref. [4]. This model allows us to study the determinants of PS-mediated assembly in a scenario of reduced computational complexity.

## A systems approach is key

**Figure 3:**
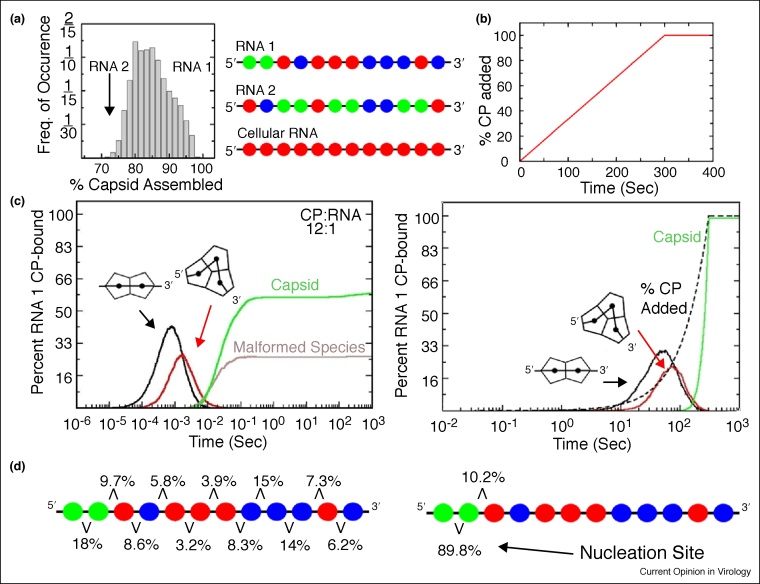
The cooperative effects of PS distributions can only be observed in the presence of the protein ramp. **(a)** Differences in the PS affinity distributions for different RNAs, that is, different bead configurations in the mathematical model, result in differences in particle yield. The spectrum of different particle yields over 30 000 random RNAs is shown, with the best (RNA1) and worst (RNA2) performing RNA shown to the right. Cellular RNAs are modelled by strings of low affinity PSs (red beads). **(b)** In a viral infection, protein is synthesized while capsid assembly already takes place, a phenomenon known as the protein ramp. It is modelled via gradual addition of CP according to the graph shown. **(c)** The assembly of virus and malformed particles in the absence (left) and presence (right) of the protein ramp reveals the importance of the protein ramp for virion yield. In particular, in the presence of the protein ramp, assembly of RNAs (shown here for RNA1) is more efficient than in its absence, where malformed species deplete the protein resource. **(d)** Nucleation behaviour depends on the protein ramp: it is dispersed across the genome (indicated by hooks together with an indication of the percentage of sequences nucleating at any given pair of PSs) in the absence, and localized at the 5′ end in the presence of the protein ramp.

Assembly against a backdrop of cellular competitor RNAs (in a 1:300 ratio consistent with experimental studies) [38^••^] reveals relatively low yields of viral particles compared with an abundance of misencapsidated particles (Figure 3), implying that in this simple form the model would not account for the assembly efficiency expected *in vivo*. This suggests that a key feature of the assembly process *in vivo* is missing in the model. Bacteriophage Qβ assembly has been studied by Eigen and collaborators [42], demonstrating that CP concentration gradually builds up while virion assembly is taking place, a phenomenon known as the protein ramp. Therefore, instead of adding the entire aliquot of CP (corresponding to the number of CP needed to fully encapsulate all viral RNAs in the simulation) at the start, a protein ramp was built into the model that reflects the gradual build-up of CP concentration, as is the case in a viral infection *in vivo*. Under these conditions, the model outcome reflects the observed *in vivo* behaviour for MS2 and other single-stranded RNA viruses [43,44], with viral particles now being the dominant species at the end of the simulation.

These results enable an important biological conclusion. They imply that the cooperative action of the PSs in enhancing assembly efficiency is best observed in experiments that are carried out under the conditions of the protein ramp, that is, a CP titration, explaining perhaps why PSs have long been missed by *in vitro* experiments. Indeed, experiments carried out in the context of a protein ramp reveal the hallmarks of PS-mediated assembly in a model virus, demonstrating that both the spacing between PSs and their recognition motifs impact on virion assembly [45^••^].

## A solution to a viral-equivalent of Levinthal’s Paradox

The model also reveals the mechanism by which viruses efficiently navigate the landscape of possible assembly intermediates [38]. In protein folding, the ensemble of potential folding pathways of an amino acid sequence into its native conformation is so complex that a random exploration of different options would take longer than the known age of the universe. Despite this, proteins fold within biologically meaningful timeframes, a phenomenon known as Levinthal’s Paradox, which we now understand, because protein chains do not sample all possible conformations on their way to their folded state. Similarly, the number of geometrically distinct ways in which a viral capsid can be built from CP is vast, yet virus assembly must have evolved strategies to bias assembly to the most efficient assembly pathways in order to sustain a productive infection against host defence mechanisms. Our model of PS-mediated assembly demonstrates how multiple dispersed PSs with varying affinities for CP can achieve this under the condition of the protein ramp (Figure 3). In particular, variations in PS affinities for CP across the genomic sequence result in nucleation of assembly at specific sites, as opposed to nonlocalised nucleation across the full length of the RNA genome in the absence of the protein ramp, that is, PSs impact on nucleation behaviour. Only a small number of distinct assembly pathways from the ensemble of geometrically possible ones are actually realized during PS-mediated assembly, which are characterized by assembly intermediates that deviate only minimally from those maximising CP:CP contacts. This demonstrates that the PS distribution mitigates the combinatorial complexity of the assembly process. In short, it solves a virus-equivalent to Levinthal’s Paradox in protein folding.

## Hamiltonian paths analysis

**Figure 4:**
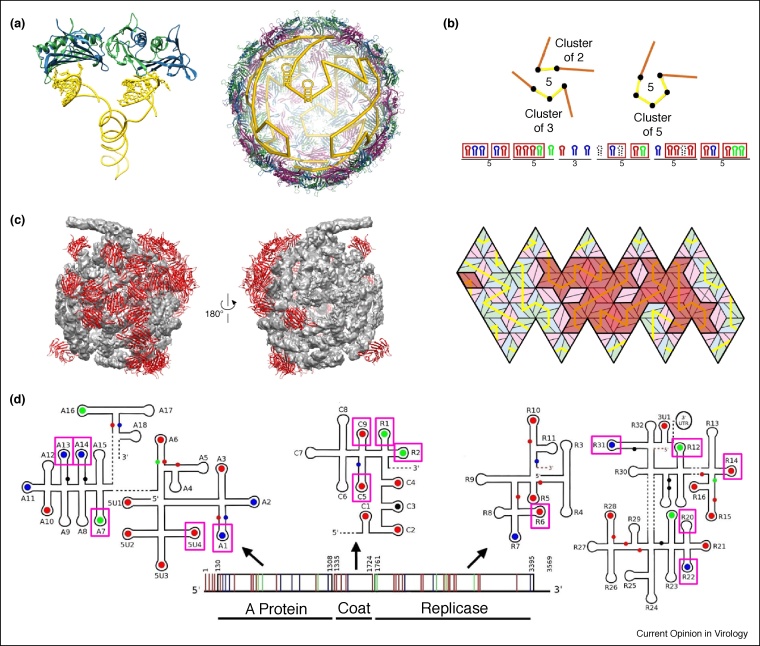
Hamiltonian Path Analysis. **(a)** Example of a Hamiltonian path in MS2, together with the 3D structure of a genomic fragment encompassing two neighbouring PSs (The stem–loops (PDB ID: 5TC1) and the backbone connecting them have been taken from the high-resolution structure in Ref. [50^••^], and the coat protein shell is shown as ribbons based on the icosahedrally averaged X-ray structure (PDB ID: 1ZDH). The example demonstrates that Hamiltonian paths are mathematical idealization of more complex RNA configurations. **(b)** A classification of all possible Hamiltonian paths for a given capsid geometry results in a set of local rules, that can be used to formulate combinatorial constraints in a bioinformatics search for PS motifs. **(c)** PSs identified in a cryo-EM reconstruction of MS2 at 8.7 Å resolution (left; adapted from Ref. [49^••^]) are located predominantly in one half of the capsid. This is in agreement with model predictions (right; based on results from Ref. [32]), showing that positions of strongly constrained PSs (PS bound to CP indicated as red rhombs) are predominantly located in one half of the capsid surface (here shown as a planar embedding of an icosahedral surface, with capsid protein dimers indicated as rhombs in colour-coding from Figure 1a). **(d)** PS positions predicted by Hamiltonian Path Analysis are shown relative to the primary and secondary structure of the MS2 genome, with green, red and blue dots or lines representing PS with high, intermediate and low affinity for capsid protein. All 15 PSs identified in a cryo-EM reconstruction of MS2 at 3.6 Å resolution [49^••^] (boxed) have been predicted by Hamiltonian Path Analysis.

Different assembly scenarios can be encoded by geometric book-keeping devices that capture the order in which PSs make contact with CP during virus assembly. In particular, by connecting PS binding sites on the capsid interior in the order in which the corresponding PS:CP contacts are made, a connected string is obtained that provides a geometric representation of the assembly pathway. Superposition of all possible such strings results in a polyhedral shape with vertices at the PS binding sites at the capsid’s interior surface, and edges connecting vertices on neighbouring capsomers. From a mathematical point of view, each individual string corresponds to a Hamiltonian path on this polyhedron, that is, a connected path visiting every polyhedral vertex precisely once. They do not represent, however, the exact location of the viral genome, which can make excursions into the capsid interior (Figure 4a). The (local) geometric properties of these paths can be classified for different types of capsid geometries. These local properties of the paths (as illustrated in Figure 4b for MS2) can then be used, in combination with a bioinformatics search for potential PS candidates, to identify the likely PS distribution [32,46,47^••^]. Note that it is not necessary for all binding sites to be occupied, and that the Hamiltonian path constraints can be more restrictive in some regions of the genome than in others. For example, our Hamiltonian Paths Analysis predicted PSs for bacteriophage MS2, that are in excellent agreement with the RNA:CP binding sites identified via cross-linking immunoprecipitation (CLIP) experiments [48^••^]. Our analysis shows that PSs are more constrained in one half of the MS2 capsid (see red rhombs in Figure 4c based on Ref. [32]), which agrees well with an asymmetric EM reconstruction of MS2 at 8.7 Å resolution [49^••^]. Moreover, all PSs identified in a subsequent EM reconstruction at 3.6 Å resolution [50^••^] had previously been identified via our Hamiltonian Path Analysis method (Figure 4d). This demonstrates the utility of mathematical tools in identifying salient features in the organization of a packaged viral genome.

## Conclusions

Modeling of PS-mediated assembly demonstrates the distinct advantages of PSs for efficient capsid formation. As PS-mediated assembly confers fitness advantages to viral particles assembling via this mechanism, it is likely that it is widespread in nature. The discovery of PSs in a number of viral families infecting different hosts including humans supports this hypothesis. Even Hepatitis B virus, a DNA virus, has been shown to reveal packaging signals in its pregenomic RNA, that impact on capsid geometry by biasing assembly towards formation of *T* = 4 shells [36^•^]. It is likely that multiple dispersed PSs will be discovered in many more viral systems over the next decade, for example, in the alphaviruses [51]. Similar assembly mechanisms may even occur more widely in nature, for example in the assembly of repurposed Gag-like proteins [52^•^] with roles in intercellular RNA transfer across synaptic boutons [53^•^].

The models of PS-mediated assembly have provided mechanistic insights that could not have been obtained via experiment alone. They revealed that hallmarks of PS-mediated assembly can only be observed in the context of scenarios reflecting *in vivo* infections, and demonstrated the importance of the PS affinity distribution for efficient capsid formation. The Hamiltonian path approach has moreover served as a tool for the identification of PSs [32]. The discovery of PS-mediated assembly has opened up novel opportunities for anti-viral therapy, for example, via small molecular weight compounds blocking either the PS or CP sites of the PS:CP interactions. The modelling paradigm reviewed here provides a basis for the study of viral infections and viral evolution, and such models have been constructed in order to study the merits of different anti-viral strategies [54^•^] and the resilience of PS-mediated assembly under mutational pressures [55^•^]. The detailed understanding of the characteristics and functional roles of the PS distribution has moreover enabled novel applications in bionanotechnology. The PS assembly code can be isolated and repurposed for the construction of stable virus-like particles with improved assembly efficiency compared with their viral counterparts, as demonstrated for Satellite Tobacco Necrosis Virus [56^••^]. Such particles might be used as decoys, gene delivery vectors, or for vaccination purposes.

## References and recommended reading

Papers of particular interest, published within the period of review, have been highlighted as

• of special interest
•• of outstanding interest

## References

[1] Nguyen H.D., Reddy V.S., Brooks C.L. (2007). Deciphering the kinetic mechanism of spontaneous self-assembly of icosahedral capsids. Nano Lett.

[2] Endres D., Zlotnick A. (2002). Model-based analysis of assembly kinetics for virus capsids or other spherical polymers. Biophys J.

[3] Zlotnick A. (2005). Theoretical aspects of virus capsid assembly. J Mol Recognit.

[4] Zlotnick A. (1994). To build a virus capsid. J Mol Biol.

[5] Bruinsma R.F., Gelbart W.M., Reguera D., Rudnick J., Zandi R. (2003). Viral self-assembly as a thermodynamic process. Phys Rev Lett.

[6] Reddy V.S., Giesing H.A., Morton R.T., Kumar A., Post C.B., Brooks C.L., Johnson J.E. (1998). Energetics of quasiequivalence: computational analysis of protein–protein interactions in icosahedral viruses. Biophys J.

[7] Zandi R., Reguera D., Bruinsma R.F., Gelbart W.M., Rudnick J. (2004). Origin of icosahedral symmetry in viruses. Proc Natl Acad Sci.

[8] Berger B., Shor P.W., Tucker-Kellogg L., King J. (1994). Local rule-based theory of virus shell assembly. Proc Natl Acad Sci U S A.

[9] Schwartz R., Shor P.W., Prevelige P.E., Berger B. (1998). Local rules simulation of the kinetics of virus capsid self-assembly. Biophys J.

[10] Borodavka A., Tuma R., Stockley P.G. (2012). Evidence that viral RNAs have evolved for efficient, two-stage packaging. Proc Natl Acad Sci.

[11•] Tubiana L., Božič A.L., Micheletti C., Podgornik R. (2015). Synonymous mutations reduce genome compactness in icosahedral ssRNA viruses. Biophys J.

[12] Gopal A., Egecioglu D.E., Yoffe A.M., Ben-Shaul A., Rao A.L.N., Knobler C.M., Gelbart W.M. (2014). Viral RNAs are unusually compact. PLoS One.

[13•] Garmann R.F., Comas-Garcia M., Knobler C.M., Gelbart W.M. (2016). Physical principles in the self-assembly of a simple spherical virus. Acc Chem Res.

[14•] Kelly J., Grosberg A.Y., Bruinsma R. (2016). Sequence dependence of viral RNA encapsidation. J Phys Chem B.

[15] Van Der Schoot P., Bruinsma R. (2005). Electrostatics and the assembly of an RNA virus. Phys Rev E — Stat Nonlinear Soft Matter Phys.

[16] Van Der Schoot P., Zandi R. (2007). Kinetic theory of virus capsid assembly. Phys Biol.

[17] Erdemci-Tandogan G., Wagner J., Van Der Schoot P., Podgornik R., Zandi R. (2014). RNA topology remolds electrostatic stabilization of viruses. Phys Rev E — Stat Nonlinear Soft Matter Phys.

[18] Garmann R.F., Comas-Garcia M., Koay M.S.T., Cornelissen J.J.L.M., Knobler C.M., Gelbart W.M. (2014). Role of electrostatics in the assembly pathway of a single-stranded RNA virus. J Virol.

[19••] Erdemci-Tandogan G., Orland H., Zandi R. (2017). RNA base pairing determines the conformations of RNA inside spherical viruses. Phys Rev Lett.

[20•] Li S., Erdemci-Tandogan G., Van Der Schoot P., Zandi R. (2018). The effect of RNA stiffness on the self-assembly of virus particles. J Phys Condens Matter.

[21] Beren C., Dreesens L.L., Liu K.N., Knobler C.M., Gelbart W.M. (2017). The effect of RNA secondary structure on the self-assembly of viral capsids. Biophys J.

[22••] Kegel W.K., Van Der Schoot P. (2006). Physical regulation of the self-assembly of tobacco mosaic virus coat protein. Biophys J.

[23••] Hagan M.F., Zandi R. (2016). Recent advances in coarse-grained modeling of virus assembly. Curr Opin Virol.

[24] Rapaport D.C. (2010). Modeling capsid self-assembly: design and analysis. Phys Biol.

[25] Chen C., Daniel M.C., Quinkert Z.T., De M., Stein B., Bowman V.D., Chipman P.R., Rotello V.M., Kao C.C., Dragnea B. (2006). Nanoparticle-templated assembly of viral protein cages. Nano Lett.

[26] Hu Y., Zandi R., Anavitarte A., Knobler C.M., Gelbart W.M. (2008). Packaging of a polymer by a viral capsid: the interplay between polymer length and capsid size. Biophys J.

[27] Garmann R.F., Sportsman R., Beren C., Manoharan V.N., Knobler C.M., Gelbart W.M. (2015). A simple RNA–DNA scaffold templates the assembly of monofunctional virus-like particles. J Am Chem Soc.

[28] Valegård K., Murray J.B., Stockley P.G., Stonehouse N.J., Liljas L. (1994). Crystal structure of an RNA bacteriophage coat protein–operator complex. Nature.

[29] Stockley P.G., Rolfsson O., Thompson G.S., Basnak G., Francese S., Stonehouse N.J., Homans S.W., Ashcroft A.E. (2007). A simple, RNA-mediated allosteric switch controls the pathway to formation of a *T* = 3 viral capsid. J Mol Biol.

[30] Dykeman E.C., Twarock R. (2010). All-atom normal-mode analysis reveals an RNA-induced allostery in a bacteriophage coat protein. Phys Rev E — Stat Nonlinear Soft Matter Phys.

[31] Dykeman E.C., Stockley P.G., Twarock R. (2010). Dynamic allostery controls coat protein conformer switching during MS2 phage assembly. J Mol Biol.

[32] Dykeman E.C., Stockley P.G., Twarock R. (2013). Packaging signals in two single-stranded RNA viruses imply a conserved assembly mechanism and geometry of the packaged genome. J Mol Biol.

[33] Dykeman E.C., Grayson N.E., Toropova K., Ranson N.A., Stockley P.G., Twarock R. (2011). Simple rules for efficient assembly predict the layout of a packaged viral RNA. J Mol Biol.

[35••] Shakeel S., Dykeman E.C., White S.J., Ora A., Cockburn J.J.B., Butcher S.J., Stockley P.G., Twarock R. (2017). Genomic RNA folding mediates assembly of human parechovirus. Nat Commun.

[36•] Patel N., White S.J., Thompson R.F., Bingham R., Weiß E.U., Maskell D.P., Zlotnick A., Dykeman E.C., Tuma R., Twarock R. (2017). HBV RNA pre-genome encodes specific motifs that mediate interactions with the viral core protein that promote nucleocapsid assembly Nikesh. Nat Microbiol.

[37] Dykeman E.C., Stockley P.G., Twarock R. (2013). Building a viral capsid in the presence of genomic RNA. Phys Rev E — Stat Nonlinear Soft Matter Phys.

[38••] Dykeman E.C., Stockley P.G., Twarock R. (2014). Solving a Levinthal’s paradox for virus assembly identifies a unique antiviral strategy. Proc Natl Acad Sci U S A.

[39] Caspar D.L.D., Klug A. (1962). Physical principles in the construction of regular viruses. Cold Spring Harb Symp Quant Biol.

[40] Lago H., Fonseca S.A., Murray J.B., Stonehouse N.J., Stockley P.G. (1998). Dissecting the key recognition features of the MS2 bacteriophage translational repression complex. Nucleic Acids Res.

[41] Poudel L., Twarock R., Steinmetz N.F., Podgornik R., Ching W.Y. (2017). Impact of hydrogen bonding in the binding site between capsid protein and MS2 bacteriophage ssRNA. J Phys Chem B.

[42] Eigen M. (2000). Viruses: evolution, propagation, and defense. Nutr Rev.

[43] Routh A., Domitrovic T., Johnson J.E. (2012). Host RNAs, including transposons, are encapsidated by a eukaryotic single-stranded RNA virus. Proc Natl Acad Sci.

[44] Routh A., Domitrovic T., Johnson J.E. (2012). Packaging host RNAs in small RNA viruses: an inevitable consequence of an error-prone polymerase?. Cell Cycle.

[45••] Patel N., Dykeman E.C., Coutts R.H., Lomonossoff G.P., Rowlands D.J., Phillips S.E.V., Ranson N., Twarock R., Tuma R., Stockley P.G. (2015). Revealing the density of encoded functions in a viral RNA. Proc Natl Acad Sci.

[46] Prevelige P.E. (2016). Follow the yellow brick road: a paradigm shift in virus assembly. J Mol Biol.

[47••] Twarock R., Leonov G., Stockley P.G. (2018). Hamiltonian path analysis of viral genomes. Nat Comms.

[48••] Rolfsson Ó, Middleton S., Manfield I.W., White S.J., Fan B., Vaughan R., Ranson N.A., Dykeman E., Twarock R., Ford J. (2016). Direct evidence for packaging signal-mediated assembly of bacteriophage MS2. J Mol Biol.

[49••] Koning R.I., Gomez-Blanco J., Akopjana I., Vargas J., Kazaks A., Tars K., Carazo J.M., Koster A.J. (2016). Asymmetric cryo-EM reconstruction of phage MS2 reveals genome structure in situ. Nat Commun.

[50••] Dai X., Li Z., Lai M., Shu S., Du Y., Zhou Z.H., Sun R. (2016). In situ structures of the genome and genome-delivery apparatus in a single-stranded RNA virus. Nature.

[51] Mendes A., Kuhn R. (2018). Alphavirus nucleocapsid packaging and assembly. Viruses.

[52•] Ashley J., Cordy B., Lucia D., Fradkin L.G., Budnik V., Thomson T. (2018). Retrovirus-like Gag protein Arc1 binds RNA and traffics across synaptic boutons. Cell.

[53•] Pastuzyn E.D., Day C.E., Kearns R.B., Kyrke-Smith M., Taibi A.V., McCormick J., Yoder N., Belnap D.M., Erlendsson S., Morado D.R. (2018). The neuronal gene arc encodes a repurposed retrotransposon gag protein that mediates intercellular RNA transfer. Cell.

[54•] Bingham R.J., Dykeman E.C., Twarock R. (2017). RNA virus evolution via a quasispecies-based model reveals a drug target with a high barrier to resistance. Viruses.

[55•] Dykeman E.C. (2017). A model for viral assembly around an explicit RNA sequence generates an implicit fitness landscape. Biophys J.

[56••] Patel N., Wroblewski E., Leonov G., Phillips S.E.V., Tuma R., Twarock R., Stockley P.G. (2017). Rewriting nature’s assembly manual for a ssRNA virus. Proc Natl Acad Sci.

